# Aminopeptidasic Enzymes as Early Biomarkers of Cardiac Surgery-Associated Acute Kidney Injury and Long-Term Events

**DOI:** 10.3390/biom14091049

**Published:** 2024-08-24

**Authors:** Noelia Rísquez Chica, Elisa Pereira, Francisco Manzano, María Mar Jiménez Quintana, Antonio Osuna, María Carmen Ruiz Fuentes, Rosemary Wangensteen

**Affiliations:** 1 Nephrology Unit, Virgen de las Nieves University Hospital, FIBAO, Instituto de Investigación Biosanitaria ibs, 18014 Granada, Spain; noelia.rc.9295@mail.com (N.R.C.); elisapereirap@gmail.com (E.P.); aosunaortega@ugr.es (A.O.); 2 Intensive Care Unit, Virgen de las Nieves University Hospital, FIBAO, Instituto de Investigación Biosanitaria ibs, 18014 Granada, Spain; franciscol.manzano.sspa@juntadeandalucia.es (F.M.); mariam.jimenez.sspa@juntadeandalucia.es (M.M.J.Q.); 3 Area of Physiology, Department of Health Sciences, University of Jaén, 23071 Jaén, Spain

**Keywords:** acute kidney injury, aminopeptidases, biomarkers, tubular lesions, glomerular filtration rate, renal replacement therapy

## Abstract

Background: Diagnosis of acute kidney injury (AKI) relies on serum creatinine (SCr) changes. This study investigated if urinary aminopeptidases are early and predictive biomarkers of cardiac surgery-associated AKI (CSA-AKI). Methods: Glutamyl aminopeptidase (GluAp), alanyl aminopeptidase (AlaAp), dipeptidyl peptidase-4 (DPP4), proteinuria, albuminuria, N-acetyl-β-*D*-glucosaminidase (NAG), and neutrophile gelatinase-associated lipocalin (NGAL) were measured in urine samples from 44 patients at arrival in the intensive care unit (ICU) after cardiac surgery. Sensitivity, specificity, and positive and negative predictive value for diagnosis of stages 1, 2, and 3 of AKI were analyzed for the highest quartile of each marker. We also studied the relationship with SCr after surgery, 6- and 12-month glomerular filtration rates (GFRs), and other long-term events over the next 5 years. Results: GluAp diagnosed the maximal number of patients that developed stage 2 or 3 of AKI, increasing diagnostic sensitivity from 0% to 75%. In addition, GluAp and DPP4 were related to the decrease in GFR at 6 or 12 months after surgery. Conclusions: Urinary aminopeptidases are a potential tool for the early diagnosis of CSA-AKI, with GluAp being the most effective marker for diagnosing stage 2 or 3 of AKI at ICU admission. GluAp and DPP4 serve as predictive biomarkers for a decrease in GFR.

## 1. Introduction

Acute kidney injury (AKI) is the most common major complication of cardiac surgery [1]. Each year, more than 2 million cardiac surgeries are performed worldwide [2], and the incidence of cardiac surgery-associated AKI (CSA-AKI) varies from 5% to 42% [3]. CSA-AKI is the second most common cause of AKI after sepsis in the intensive care setting and is independently associated with increased morbidity and mortality [4]. CSA-AKI is characterized by an abrupt deterioration in kidney function, which is evidenced by a reduction in glomerular filtration rate (GFR) [4].

Criteria for diagnosing AKI have been based on the Acute Kidney Injury Network (AKIN) and/or the Risk, Injury, Failure, Loss, End Stage Kidney Disease (RIFLE) criteria to define AKI. These criteria are based on serum creatinine (SCr) changes (SCr ≥ 0.3 mg/dL or 26.5 µmol/L within 48 h, or SCr increase by at least 1.5 times baseline) and urine output (<0.5 mL/kg/h for at least 6 h) [5]. According to the Kidney Disease Improving Global Outcomes (KDIGO) organization, patients that fulfill these criteria are considered to be in stage 1 of AKI [6]. Unfortunately, in patients who have undergone cardiac surgery, the application of these criteria is controversial, because fluid resuscitation and fluid loading from the pump priming step during cardiopulmonary bypass are universal. Thus, SCr changes owing to fluid balance can lead to AKI being underdiagnosed [7]. Furthermore, the RIFLE criteria classify all patients who have undergone renal replacement therapy (RRT) as being in the ‘failure’ stage, but the criteria used to initiate RRT are not standardized [8].

SCr level is traditionally used as a biomarker for renal impairment, but it can be affected not only by physiological processes (for example, urinary clearance of creatinine, or muscle mass) but also by drugs that block the tubular secretion of creatinine and by some underlying medical conditions, such as diabetes and liver disease [8]. Furthermore, SCr is considered by some authors as a delayed biomarker because it rises when renal dysfunction is patent, and it cannot distinguish the etiology of the lesion [9].

Although SCr concentration is useful in later diagnosis and prognosis of CSA-AKI, early detection of acute renal damage remains challenging, and there is a need to identify new biomarkers that can contribute to enhancing clinical outcomes and treatments [10].

Injured tubular cells excrete enzymes and other proteins in the urine that are potential biomarkers for the early detection of renal disease [11]. These cells contain aminopeptidases, mainly alanyl aminopeptidase (AlaAp) and glutamyl aminopeptidase (GluAp), that have been described as early and predictive urinary biomarkers of renal injury severity in different animal models [12,13,14].

Dipeptidyl peptidase-4 (DPP4), also called CD26, is a serine protease membrane glycoprotein with exopeptidase activity [15]. DPP4 is expressed on the surface of several cell types, including epithelial cells, endothelial cells, and lymphocytes [15,16]. This peptidase also exists in a soluble circulating form (sDPP4) in plasma and other body fluids [17]. Strong expression of DPP4 has been observed in the kidney [16], predominantly in the glomeruli and S1–S3 segments of the nephron [18], as well as in vascular endothelial cells, suggesting that this protein may play a role in renal and cardiovascular function [19], as well as in diabetes, hypertension, and chronic kidney disease progression [18].

This work aimed to investigate if the determination of GluAp, AlaAp, and/or DPP4 in urine collected at admission in the intensive care unit (ICU) could be useful in the early diagnosis of CSA-AKI, comparing their diagnostic sensitivity and specificity with AKIN criteria and other urinary markers such as proteinuria, albuminuria, NAG, and NGAL. In addition, we analyzed their relationship with the need for RRT, the length of stay in ICU, and the glomerular filtration rate (GFR) determined 6 and 12 months after surgery, as well as with subsequent hospitalizations, cardiovascular events, and exitus in the following 5 years.

## 2. Materials and Methods

### 2.1. Study Design

In total, 44 patients who had undergone cardiac surgery from 4 March 2015 to 29 June 2015 were selected for the study. Inclusion criterion: cardiac surgery with cardiopulmonary bypass. Exclusion criterion: previous history of renal dysfunction. Patients showed a basal SCr of 0.92 ± 0.03 (mean ± SEM) and a basal GFR of 74.0 ± 2.93 (mean ± SEM). Overall, 20% of patients were subjected to coronary revascularization, 2% were subjected to the closure of interatrial communication, and 78% were subjected to valvular surgery (57.1% aortic valve, 25.7 mitral valve, 14.3 mitral/aortic valve, and 2.9% mitral/aortic/tricuspid valve). Regarding comorbidities, 67% of patients displayed arterial hypertension, 31.3% diabetes mellitus, 40% ischemic heart disease, 6.7% peripheral vascular disease, and 15.6% cerebrovascular disease. This study was conducted in accordance with the Declaration of Helsinki and approved by the Ethics Committee of Virgen de las Nieves University Hospital with approval ID PI-0668-2013 on 1 May 2013.

Blood samples were taken before surgery, at admission to the ICU, and at 12 h, 48 h, 6 months, and 12 months after surgery. Urine samples were collected at admission to the ICU. SCr was measured in blood samples. GFR was calculated at 6 and 12 months after surgery using the CKD-EPI equation [20]. Creatinine, proteinuria, albuminuria, GluAp, AlaAp, DPP4, N-acetyl-β-D-glucosaminidase (NAG) activity, and neutrophil gelatinase-associated lipocalin (NGAL) were measured in all urine samples at admission to the ICU.

SCr values were used to classify patients in their corresponding stage of AKI: stage 1 (increased creatinine of ≥0.3 mg/dL or increase to ≥150–200% from baseline); stage 2 (increased creatinine from >200–300%), and stage 3 (increased creatinine to >300% from or SCr ≥ 4 mg/dL). For statistical purposes, patients classified as stage 3 were included in the same group as stage 2 throughout the study.

### 2.2. Analytical Procedures

Blood samples were centrifuged for 15 min at 1000× *g*, and SCr was measured immediately in the Clinical Laboratory Unit of the hospital using an autoanalyzer Alinity C from Abbott that uses the creatinine-Jaffé method based on the kinetics of reaction of serum creatinine with alkaline picrate (ref. 04T9120).

Urine samples were centrifuged at 1000× *g* for 15 min and frozen at −80 °C until analysis. No special additives were added.

Urinary creatinine, proteinuria, albuminuria, and NAG activity were determined by an autoanalyzer Spin120. Reagents for proteinuria (ref. MI1001025), albuminuria (ref. 1107170), and the creatinine-Jaffé method (ref. MI1001111) were provided by Spinreact (Barcelona, Spain). The reagent for NAG (ref. DZ062A-K) was purchased from Dyazyme Laboratories (Poway, CA, USA). GluAp was measured in urine samples using a commercial ELISA kit purchased from GeneBio Systems (Burlington, ON, Canada). DPP4 was measured in urine samples using a commercial ELISA kit purchased from Assaypro (St. Charles, MO, USA). AlaAp and NGAL were measured with an ELISA kit from Boster Bio (Pleasanton, CA, USA). All analytes were measured in duplicate.

### 2.3. Statistical Analysis

We used the *t* test for analysis of continuous variables with normal distribution and the Mann–Whitney U (Wilcoxon) test when data did not correspond to a normal distribution. Simple ANOVA with Bonferroni’s test or the Kruskal–Wallis were used when comparing more than two groups. Differences were considered statistically significant at the *p* < 0.05 level.

Comparison of different rates between groups was carried out using the χ^2^ test. Differences were considered statistically significant at the *p* < 0.05 level.

Factorial ANOVA for paired data was used to compare the evolution of SCr and GFR, taking AKI as the inter-subject factor and each time point as the intra-subject factor. Differences were analyzed with Bonferroni’s test and were considered statistically significant at the *p* < 0.05 level. The same analysis was used to compare the evolution of SCr and GFR, taking the highest quartile of each marker as the inter-subject factor. Differences were considered statistically significant at the *p* < 0.05 level.

All statistical analysis were carried out with StatGraphics 19 ^®^.

## 3. Results

### 3.1. Demographic Characteristics and Long-Term Events in Patients That Developed AKI and Patients That Did Not Develop AKI after Cardiac Surgery

In total, 43.2% of patients (*n* = 19) developed AKI after surgery. AKI patients were found to be significantly older than patients that did not develop AKI, although no differences were found in gender distribution (Table 1). Patients that developed AKI displayed higher percentages of needing renal replacement therapy and exitus, but there were not any significant differences in the length of stay in the ICU after surgery, subsequent hospitalizations, or cardiovascular events.

Overall, 25% of patients (*n* = 11) were diagnosed with stage 1 AKI, 11.4% of patients (*n* = 5) developed AKI stage 2, and 6.82% (*n* = 3) of patients developed AKI stage 3. Patients in stage 2 or 3 (*n* = 8) were treated as the same group for statistical purposes. In this group, a significant percentage of patients needed RRT, but there were no significant differences in the rest of the long-term events (Table 2).

### 3.2. Evolution of SCr after Cardiac Surgery

**Figure 1:**
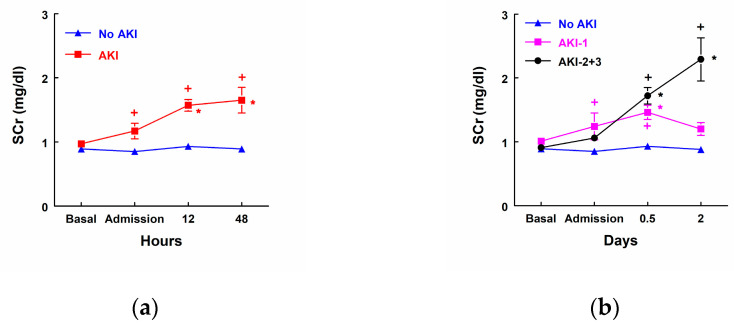
Serum creatinine (SCr) concentration (mg/dL) before surgery, at admission to the intensive care unit (ICU), and 12 h and 48 h after surgery in (**a**) patients that did not develop AKI (No AKI, *n* = 25) and patients that developed AKI (*n* = 19), as well as (**b**) patients that did not develop AKI (No AKI, *n* = 25), patients that developed stage 1 AKI (AKI-1, *n* = 11), and patients that developed stage 2 or 3 AKI (AKI-2 + 3, *n* = 8). Means ± SEM are displayed. * *p* < 0.05 vs. patients at their own basal level. + *p* < 0.05 vs. patients that did not develop AKI.

AKI patients (*n* = 19) showed a significant increase in SCr concentrations at admission to the ICU 12 and 48 h after surgery when compared with patients that did not develop AKI (No AKI, *n* = 25). SCr was also increased 12 and 48 h after surgery when it was compared to their own basal levels (Figure 1a). SCr concentration was significantly increased 12 and 48 h after surgery in patients that developed stage 2 or 3 AKI (*n* = 8). Nevertheless, patients in stage 1 AKI (*n* = 11) showed a transient increase in SCr at admission to the ICU and 12 h after surgery, but there were not any significant differences 48 h after surgery in comparison with their own basal levels or with patients that did not develop AKI (Figure 1b).

### 3.3. Evolution of Glomerular Filtration Rate

**Figure 2:**
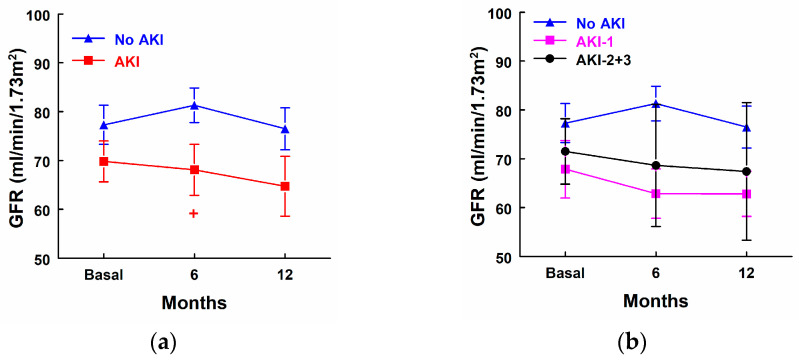
Glomerular filtration rate (GFR) before surgery, 6 months after surgery, and 12 months after surgery in (**a**) patients that did not develop AKI (*n* = 23) and patients that developed AKI (*n* = 12), as well as (**b**) patients that did not develop AKI (No AKI, *n* = 23), patients that developed stage 1 AKI (AKI-1, *n* = 7), and patients that developed stage 2 or 3 AKI (AKI-2 + 3, *n* = 5). + *p* < 0.05 vs. patients that did not develop AKI. Means ± SEM are displayed. + *p* < 0.05 vs. patients that did not develop AKI.

AKI patients showed a significant decrease in GFR 6 months after surgery in comparison with patients that did not develop AKI, but there were not any statistical differences in GFR in comparison with their own basal levels in any group of patients (Figure 2a). GFR was decreased in AKI-1 and AKI-2 + 3 at 6 and 12 months after surgery, but these differences did not reach significance (Figure 2b).

### 3.4. Urinary Markers at Admission to the ICU

To analyze if urinary markers can contribute to early diagnosis of AKI, proteinuria, albuminuria, NAG, NGAL, GluAp, AlaAp, and DPP4 were measured in urine samples obtained at admission to the ICU. These results are displayed in Table 3 and Table 4.

Albuminuria was the only marker significantly increased in the urine of AKI patients at admission to the ICU in comparison with patients that did not develop AKI (Table 3). Patients that developed stage 2 or 3 AKI displayed higher excretion of GluAp and NAG activity at admission to the ICU in comparison with No AKI or AKI-1 patients. The other tubular enzymes, AlaAp and DPP4, were also increased in urine, but the differences were not statistically significant (Table 4).

### 3.5. Sensitivity and Specificity of Urinary Biomarkers at Admission to the ICU

Cut-off value (COV), positive predictive value (PPV, %), negative predictive value (NPV, %), sensitivity (Sens, %), specificity (Spec, %), and percentage of patients correctly diagnosed with AKI (CDP) were calculated for the highest quartile of each biomarker at admission to the ICU. Albuminuria, NAG, GluAp, and DPP4 could correctly diagnose 63.6% of patients, a greater percentage than AKIN criteria, at 61.4%, maintaining specificity over 80% and increasing sensitivity from 10.5% to 36.8% (Table 5).

In the case of stage 2 or 3 AKI, GluAp was the marker that served to correctly diagnose a higher percentage of patients at admission to the ICU (84.1%), showing a sensitivity of 75% with 86.1% specificity and the highest positive (54.5%) and negative (93.9%) predictive values (Table 6). On the contrary, AKIN criteria were not useful to diagnose any patient with stage 2 or 3 AKI at admission to the ICU, because none of these patients displayed more than a 200% of increase in SCr at this point. Therefore, determination of urinary GluAp at admission to the ICU is a better diagnostic tool than other urinary markers or even AKIN criteria for stage 2 or 3 AKI.

### 3.6. Urinary Markers and Evolution of SCr after Cardiac Surgery

**Figure 3:**
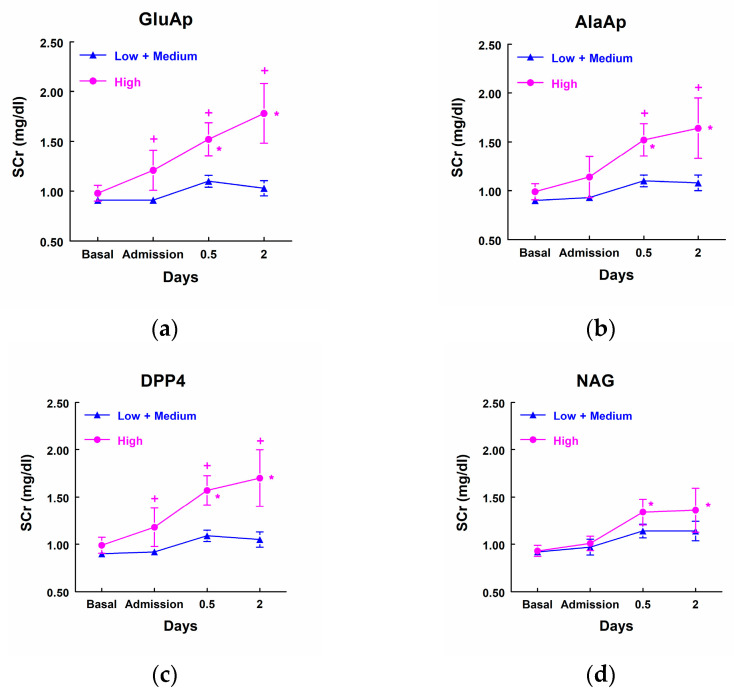
Concentration of SCr (mg/dL) in patients that showed high levels of GluAp (**a**), AlaAp (**b**), DPP4 (**c**), and NAG (**d**) at admission to the ICU (highest quartile, *n* = 11) and patients with low or medium levels of these markers (*n* = 33). Means ± SEM are displayed. * *p* < 0.05 vs. their own basal level. + *p* < 0.05 vs. the corresponding Low + Medium cluster.

Patients that showed higher excretion of GluAp, AlaAp, DPP4, and NAG at admission to the ICU, included in the highest quartile of each marker, displayed a significant increase in SCr at different points after surgery in comparison not only with their own basal level of SCr but also with patients that excreted low levels of these tubular enzymes (Figure 3). Furthermore, patients with low levels of these three enzymes did not show a significant increase in SCr at any time point after surgery.

**Figure 4:**
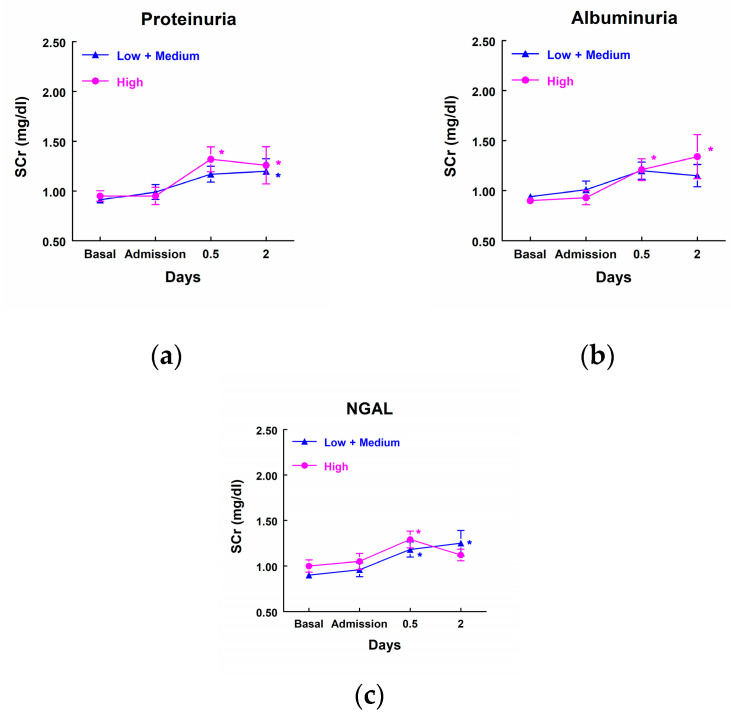
Concentration of SCr (mg/dL) in patients that showed high levels of proteinuria (**a**), albuminuria (**b**), and NGAL (**c**) at admission to the ICU (highest quartile, *n* = 11) and patients with low or medium levels of these markers (*n* = 33). Means ± SEM are displayed. * *p* < 0.05 vs. their own basal level.

On the contrary, patients with high levels of proteinuria, albuminuria, or NGAL did not show any significant increase in SCr when compared with the group of patients that excreted low levels of these markers. Furthermore, patients that excreted low or medium levels of proteinuria and NGAL showed a significant increase in SCr in comparison with their own basal level (Figure 4).

### 3.7. Urinary Biomarkers and Evolution of GFR

**Figure 5:**
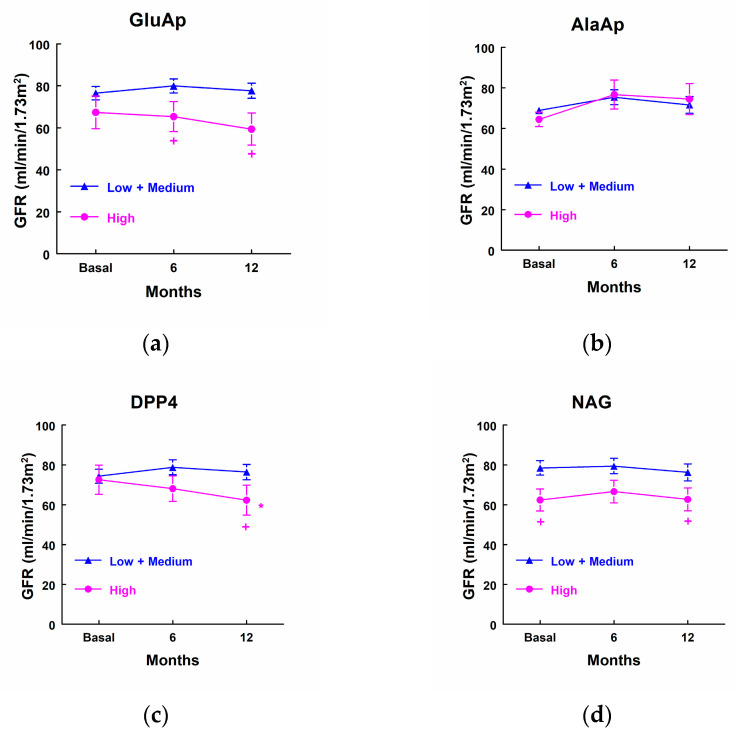
Glomerular filtration rate (mL/min/1.73 m^2^) in patients that showed high levels of GluAp (**a**), AlaAp (**b**), DPP4 (**c**), and NAG (**d**) at admission to the ICU (highest quartile, *n* = 10) and patients with low or medium levels of these markers (*n* = 25). Means ± SEM are displayed. * *p* < 0.05 vs. their own basal level. + *p* < 0.05 vs. the corresponding Low + Medium cluster.

Patients that showed higher excretion of GluAp at admission to the ICU displayed a significant decrease in GFR determined 6 and 12 months after surgery in comparison with patients that excreted low levels of GluAp (Figure 5a). In addition, patients with higher levels of DPP4 showed a significant decrease in GFR 12 months after surgery in comparison not only with patients that excreted low levels of DPP4, but also compared with their own basal levels (Figure 5c). We also observed that patients that excreted higher levels of NAG displayed decreased GFRs before surgery and 12 months after surgery when compared with patients that excreted lower levels of NAG at admission to the ICU. GFR was not decreased in comparison with their basal levels, because it was already decreased before surgery in these patients (Figure 5c).

**Figure 6:**
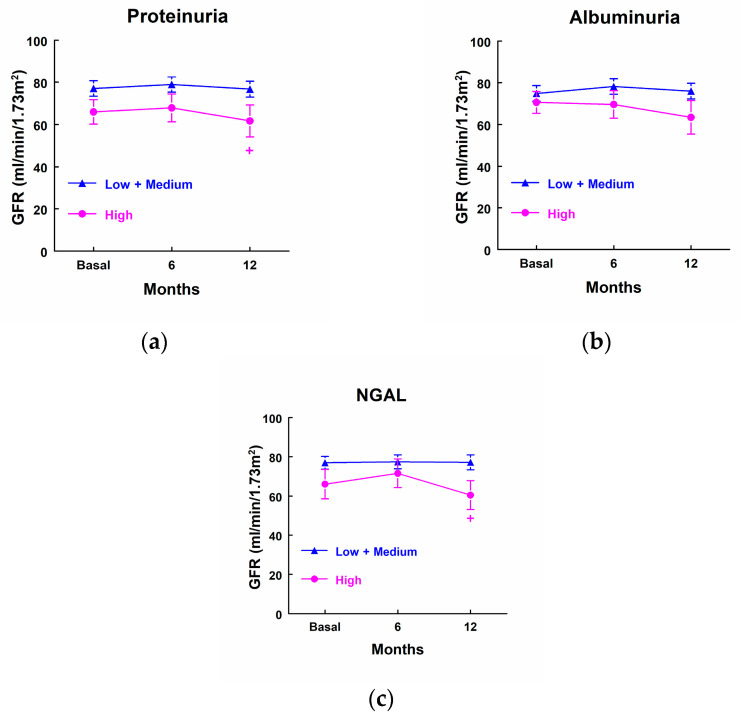
Glomerular filtration rate (mL/min/1.73 m^2^) in patients that showed high levels of proteinuria (**a**), albuminuria (**b**), and NGAL (**c**) at admission to the ICU (highest quartile, *n* = 10) and patients with low or medium levels of these markers (*n* = 25). Means ± SEM are displayed. + *p* < 0.05 vs. the corresponding Low + Medium cluster.

Patients that exhibited higher levels of proteinuria and NGAL at admission to the ICU displayed a decreased GFR 12 months after surgery when compared with patients that excreted lower levels of both markers at admission to the ICU, but it was not decreased in comparison with their own basal levels. We did not find any significant difference in GFR at any point in the case of albuminuria (Figure 6).

### 3.8. Urinary Biomarkers and Long-Term Events

There were not any significant differences in the length of stay in ICU (Table 7), subsequent hospitalizations (Table 8), need for RRT (Table 9), cardiovascular events (Table 10), or exitus (Table 11) among patients that displayed higher levels of the markers at admission to the ICU and the rest of the patients. Nevertheless, it is remarkable that the percentage of patients that needed RRT was six-fold higher (Table 9) in the case of patients that excreted higher levels of GluAp at admission to the ICU, and the percentage of exitus was three-fold higher (Table 11) than patients that exhibited low levels of GluAp at admission to the ICU.

## 4. Discussion

The main finding from this study is that GluAp, AlaAp, and DPP4 are early biomarkers of cardiac surgery-associated AKI. Furthermore, the determination of GluAp in urine collected at admission to the ICU just after surgery increased diagnostic sensitivity for stage 2 or 3 AKI compared with other urinary markers or with AKIN criteria, increasing the total number of correctly diagnosed patients. In addition, the excretion of GluAp and DPP4 at admission to the ICU was found to be related to the decrease in GFR 6 and/or 12 months after surgery.

GluAp, AlaAp, and DPP4 are enzymes that are present in brush border cells in the proximal renal tubules [21,22,23,24]. Therefore, acute kidney injury would evoke apoptosis and/or necrosis of these cells and higher excretion in the urine of these tubular proteins. Accordingly, Westhuyzen et al. [25] reported that urinary NAG levels, in addition to other tubular enzymes, were highly sensitive in detecting AKI in a population of critically ill adult patients, preceding increases in SCr. The usefulness of GluAp and AlaAp as early biomarkers was demonstrated in our laboratory in cisplatin-treated rats, an experimental model of AKI [12,13]. In this previous study, GluAp and AlaAp urinary enzymatic activities were found to be increased 24 h after cisplatin injection, and these activities could predict a rise in SCr and renal lesions in cisplatin-treated animals.

In the present work, we have investigated the role of GluAp, AlaAp, and DPP4 as biomarkers of CSA-AKI, analyzing their diagnostic sensitivity and specificity not only for patients that develop AKI at all stages, but also for patients that develop stage 2 or 3 AKI. In AKI patients, including any stage of AKI, we found that albuminuria, NAG, GluAp, and DPP4 were the markers with the greatest sensitivity (36.8%). Nevertheless, a higher sensitivity for urinary NGAL was found in other studies when it was determined 12 h after surgery [26].

Patients that develop stage 2 or 3 AKI did not show any significant differences in SCr at admission to the ICU in our study, according to findings that SCr accumulates over time and that changes in creatinine concentration become apparent only when the kidneys have lost 50% of their functional capacity [27,28]. Therefore, the finding of markers that improve the number of correctly diagnosed patients at this point has special relevance. In this way, it is remarkable that the four urinary markers that stem from the renal tubules (GluAp, AlaAp, DPP4, and NAG) were augmented at admission to the ICU in the patients that showed the highest levels of SCr at 12 or 48 h after surgery. Nevertheless, higher proteinuria, albuminuria, and NGAL levels were not found to be related to the rise in SCr. These findings demonstrate that tubular lesions induced during cardiac surgery are related with the later development of AKI, as suggested by some authors [7,29], and that tubular enzymes can be considered as early biomarkers of AKI. In addition, these results show that the probability of developing AKI is lower in patients with an intact tubular epithelium after surgery. In our study, GluAp at admission to the ICU was the tubular enzyme that exhibited the greatest diagnostic sensitivity (75%) for patients that develop stage 2 or 3 AKI, whereas AKIN criteria were not able to diagnose any patient at this point. Similarly, other authors found that SCr started to rise in AKI patients 48 h after cardiopulmonary bypass [30], but they did not find any differences in SCr 24 h after surgery. GluAp increased to 84.1% of the total number of correctly diagnosed patients in our study, maintaining a specificity of over 80% at the same time. Note that AKIN criteria display 100% specificity at admission to the ICU because these are the criteria for diagnosis of AKI, so all patients that did not develop AKI were included as true negative patients, and no other marker can reach the diagnostic specificity of the criteria that are actually used to classify the stage of AKI.

In our study, the percentage of patients that needed RRT and the percentage of exitus were significantly higher in patients that developed AKI, coinciding with the findings of Hobson et al. [3]. We also found that patients with AKI displayed a decrease in GFR 6 months after surgery, although there were not any statistical differences at 12 months. This relationship between AKI and chronic kidney disease (CKD) has been previously reported by several authors [31,32]. In addition, we analyzed if excretion of the different markers could be related to long-term events, finding a significant relationship between higher excretion of GluAp at admission to the ICU and a decrease in GFR 6 months after surgery. Several markers (GluAp, DPP4, NAG, proteinuria, and NGAL) were found to be related to a decrease in GFR 12 months after surgery. These findings demonstrate that tubular lesions are related to a decrease in renal function and the development of CKD. Therefore, the determination of these markers at different time points after surgery could be a potential tool to evaluate the progression of renal disease in these patients.

The main limitation of the study is that the percentage of patients that develop AKI in stage 2 or 3 after cardiac surgery constitutes approximately 20% of the total patients. This fact poses a clear limitation for the statistical analysis that we solved, at least in part, by comparing the higher quartile of each marker not only with the rest of quartiles but also with basal levels of SCr or GFR, yielding more reliable statistical results. Regardless, the small number of patients with some long-term events like exitus or need for RRT made it difficult to achieve statistical significance even when the percentage of patients that suffered these events was clearly higher for the highest quartile of some markers like GluAp. Another limitation of our study is that we only measured four urinary markers, in addition to aminopeptidases. We selected two traditional markers, proteinuria and albuminuria, that are the only urinary markers that in fact are measured in clinical practice for renal pathologies, as well as NAG, because its urinary levels specifically reflect the extent of tubular damage during AKI [33], and NGAL, a marker that is produced within the kidney under ischemic conditions and has been demonstrated to increase in urine before any significant changes in SCr levels [34]. The determination of more biomarkers that have been proposed as early biomarkers of AKI would be of interest to compare their sensitivity and specify with aminopeptidasic enzymes.

Future studies that include a higher number of patients who have undergone cardiac surgery should be accomplished to further investigate aminopeptidasic enzymes as markers of AKI and to analyze their predictive role for long-term events. In this way, the determination of these enzymes at different time points after cardiac surgery could serve to study the status of the tubular epithelium after surgery in these patients, because our findings demonstrate a direct relationship between excretion of tubular enzymes and a fall in renal function. In addition, the determination of these enzymes in urine could be of interest in other pathologies or events that induce AKI.

## 5. Conclusions

We conclude that urinary aminopeptidases are useful in the early diagnosis of CSA-AKI because of their high sensitivity and specificity at arrival at the ICU just after surgery. Our results also demonstrate that tubular lesions, which are quantified by these urinary markers, are present in patients that reach the highest concentrations of SCr, and that tubular lesions are related to a long-term fall in renal function. GluAp was the best marker in our study for the diagnosis of stage 2 or 3 AKI at admission to the ICU. Both GluAp and DPP4 were related to a decrease in GFR at 6 or 12 months after surgery.

## 6. Patents

Rosemary Wangensteen and Antonio Osuna are co-inventors of the patent “Aminopeptidases as markers of renal damage” granted on 5 August 2013, with publication number 2382960. There is not any other relevant declaration related to employment, consultancy, patents, products in development, or modified products.

## Figures and Tables

**Table 1:** Demographic characteristics of and long-term events in patients that developed AKI (*n* = 19) and patients that did not develop AKI (*n* = 25) after cardiac surgery.

	No AKI (*n* = 25)	AKI (*n* = 19)
Age (years)	65.6 ± 2.31	71.7 ± 1.64 *
Gender (male/female, %)	36/64	47.4/52.6
Need for RRT (%)	0	15.8 *
Stay in ICU (days)	9.25 ± 0.84	13.6 ± 2.87
Subsequent hospitalizations (%)	25	35.3
Cardiovascular events (%)	12.5	23.5
Exitus (%)	0	21.1 *

Means ± SEM or percentages are displayed. * *p* < 0.05 vs. patients that did not develop AKI.

**Table 2:** Demographic characteristics of and long-term events in patients that did not develop AKI (No AKI, *n* = 25), patients that developed stage 1 AKI (AKI-1, *n* = 11), and patients that developed stage 2 or 3 AKI (AKI-2 + 3, *n* = 8).

	No AKI (*n* = 25)	AKI-1 (*n* = 11)	AKI-2 + 3 (*n* = 8)
Age (years)	65.6 ± 2.31	72.3 ± 2.08	71.0 ± 2.80
Gender (male/female, %)	36/64	55/45	37.5/62.5
Need for RRT (%)	0	9.09	25 *
Stay in ICU (days)	9.25 ± 0.84	10.7 ± 2.03	17.6 ± 6.20
Subsequent hospitalizations (%)	25	40	28.6
Cardiovascular events (%)	12.5	20	28.6
Exitus (%)	0	18.2	25

Means ± SEM or percentages are displayed. * *p* < 0.05 vs. patients that did not develop AKI.

**Table 3:** Proteinuria (g/g Cr), albuminuria (mg/g Cr), NAG (U/g Cr), NGAL (mg/g Cr), GluAp (mg/g Cr), AlaAp (mg/g Cr), and DPP4 (mg/g Cr) in urine samples obtained at admission to the ICU in patients that did not develop AKI (*n* = 25) and in patients that developed AKI (*n* = 19).

	No AKI (*n* = 25)	AKI (*n* = 19)
Proteinuria (g/g Cr)	11.6 ± 2.04	15.8 ± 3.42
Albuminuria (mg/g Cr)	626 ± 95.8	966 ± 113 *
NAG (U/g Cr)	114 ± 23.7	211 ± 47.4
NGAL (mg/g Cr)	15.1 ± 2.09	36.8 ± 12.5
GluAp (mg/g Cr)	1.82 ± 0.80	17.8 ± 13.4
AlaAp (mg/g Cr)	265 ± 82.3	432 ± 145
DPP4 (mg/g Cr)	70.0 ± 11.5	150 ± 40.6

Means ± SEM are displayed. * *p* < 0.05 vs. patients that did not develop AKI.

**Table 4:** Proteinuria (g/g Cr), albuminuria (mg/g Cr), NAG (U/g Cr), NGAL (mg/g Cr), GluAp (mg/g Cr), AlaAp (mg/g Cr), and DPP4 (mg/g Cr) in urine samples obtained at admission to the ICU in patients that did not develop AKI (*n* = 25) and in patients that developed AKI stage 1 (*n* = 11) and stage 2 or 3 (*n* = 8).

	No AKI or AKI-1 (*n* = 36)	AKI-2 + 3 (*n* = 8)
Proteinuria (g/g Cr)	13.3 ± 2.25	13.8 ± 2.36
Albuminuria (mg/g Cr)	723 ± 81.1	979 ± 198
NAG (U/g Cr)	129 ± 21.1	277 ± 94.4 *
NGAL (mg/g Cr)	27.8 ± 6.81	9.50 ± 2.45
GluAp (mg/g Cr)	2.32 ± 1.06	37.5 ± 31.5 *
AlaAp (mg/g Cr)	261 ± 62.5	679 ± 313
DPP4 (mg/g Cr)	71.9 ± 8.43	251.4 ± 85.8

Means ± SEM are displayed. * *p* < 0.05 vs. patients that did not develop AKI.

**Table 5:** Cut-off value (COV), predictive positive value (PPV, %), predictive negative value (PNV, %), sensitivity (Sens, %), specificity (Spec, %), and percentage of correctly diagnosed patients (CDP) for diagnosis of AKI at admission to the ICU for the highest quartile of each biomarker and AKIN criteria. Patients that developed AKI were taken as true positives (*n* = 19), and No AKI patients were taken as true negatives (*n* = 25).

Marker	COV	PPV (%)	NPV (%)	Sens (%)	Spec (%)	CDP (%)
Proteinuria (g/g Cr)	16.2	54.5	60.6	31.6	80	59.1
Albuminuria (mg/g Cr)	1246	63.6	63.6	36.8	84	63.6
NAG (U/g Cr)	174	63.6	63.6	36.8	84	63.6
NGAL (mg/g Cr)	23.1	54.6	60.6	31.6	80	59.1
GluAp (mg/g Cr)	1.840	63.6	63.6	36.8	84	63.6
AlaAp (mg/g Cr)	323	54.6	60.6	31.6	80	59.1
DPP4 (mg/g Cr)	105	63.6	63.6	36.8	84	63.6
AKIN Criteria		100	59.5	10.5	100	61.4

**Table 6:** Cut-off value (COV), predictive positive value (PPV, %), predictive negative value (PNV, %), sensitivity (Sens, %), specificity (Spec, %), and percentage of correctly diagnosed patients (CDP) for diagnosis of stage 2 or 3 AKI at admission to the ICU for the highest quartile of each biomarker and AKIN criteria. Patients that developed stage 2 or 3 AKI were taken as true positives (*n* = 8). No AKI patients and patients in stage 1 AKI were taken as true negatives (*n* = 36).

Marker	COV	PPV (%)	NPV (%)	Sens (%)	Spec (%)	CDP (%)
Proteinuria (g/g Cr)	16.2	27.3	84.8	37.5	77.8	70.5
Albuminuria (mg/g Cr)	1246	36.4	87.8	50	80.6	75
NAG (U/g Cr)	174	27.3	84.8	37.5	77.8	70.5
NGAL (mg/g Cr)	23.1	0	75.8	0	69.4	56.8
GluAp (mg/g Cr)	1.840	54.5	93.9	75.0	86.1	84.1
AlaAp (mg/g Cr)	323	36.4	87.8	50	80.6	75
DPP4 (mg/g Cr)	105	45.5	90.9	62.5	83.3	79.5
NGAL (mg/g Cr)	23.1	0	75.8	0	69.4	56.8
AKIN Criteria		0	81.8	0	100	81.8

**Table 7:** Length of stay in ICU (days) in patients that showed higher levels of urinary biomarkers at admission to the ICU (*n* = 11) in comparison with patients that showed lower or medium levels of biomarkers (*n* = 33).

	Low + Medium (*n* = 33)	High (*n* = 11)
Proteinuria (g/g Cr)	9.47 ± 0.82	16.2 ± 4.65
Albuminuria (mg/g Cr)	9.56 ± 0.86	15.9 ± 4.63
NAG (U/g Cr)	11.1 ± 1.78	11.5 ± 1.58
NGAL (mg/g Cr)	11.6 ± 1.79	9.91 ± 1.47
GluAp (mg/g Cr)	9.50 ± 0.88	16.1 ± 4.57
AlaAp (mg/g Cr)	10.3 ± 1.75	13.8 ± 1.57
DPP4 (mg/g Cr)	9.38 ± 0.83	16.5 ± 4.60

**Table 8:** Subsequent hospitalizations (%) in patients that showed higher levels of urinary biomarkers at admission to the ICU (*n* = 10) in comparison with patients that showed lower or medium levels of biomarkers (*n* = 31).

	Low + Medium (*n* = 31)	High (*n* = 10)
Proteinuria (g/g Cr)	35.5	10.0
Albuminuria (mg/g Cr)	35.5	10.0
NAG (U/g Cr)	29.0	30.0
NGAL (mg/g Cr)	32.3	20.0
GluAp (mg/g Cr)	32.3	20.0
AlaAp (mg/g Cr)	25.8	40.0
DPP4 (mg/g Cr)	29.0	30.0

**Table 9:** Need for RRT (%) in patients that showed higher levels of urinary biomarkers at admission to the ICU (*n* = 11) in comparison with patients that showed lower or medium levels of biomarkers (*n* = 33).

	Low + Medium (*n* = 33)	High (*n* = 11)
Proteinuria (g/g Cr)	6.06	9.09
Albuminuria (mg/g Cr)	9.09	0
NAG (U/g Cr)	9.09	0
NGAL (mg/g Cr)	9.09	0
GluAp (mg/g Cr)	3.03	18.2
AlaAp (mg/g Cr)	6.06	9.09
DPP4 (mg/g Cr)	6.06	9.09

**Table 10:** Cardiovascular events (%) in patients that showed higher levels of urinary biomarkers at admission to the ICU (*n* = 10) in comparison with patients that showed lower or medium levels of biomarkers (*n* = 31).

	Low + Medium (*n* = 31)	High (*n* = 10)
Proteinuria (g/g Cr)	22.6	0
Albuminuria (mg/g Cr)	22.6	0
NAG (U/g Cr)	19.4	10.0
NGAL (mg/g Cr)	19.4	10.0
GluAp (mg/g Cr)	22.6	0
AlaAp (mg/g Cr)	16.1	20.0
DPP4 (mg/g Cr)	19.4	10.0

**Table 11:** Exitus (%) in patients that showed higher levels of urinary biomarkers at admission to the ICU (*n* = 11) in comparison with patients that showed lower or medium levels of biomarkers (*n* = 32).

	Low + Medium (*n* = 32)	High (*n* = 11)
Proteinuria (g/g Cr)	9.38	9.09
Albuminuria (mg/g Cr)	12.5	0
NAG (U/g Cr)	12.5	0
NGAL (mg/g Cr)	12.5	0
GluAp (mg/g Cr)	6.25	18.2
AlaAp (mg/g Cr)	9.38	9.09
DPP4 (mg/g Cr)	9.38	9.09

## Data Availability

The data presented in this study are available at: https://docs.google.com/spreadsheets/d/1vhtNR-dx3wSBHaT-heeSOrxH6gGJpkcO/edit?usp=sharing&ouid=113469746811828789402&rtpof=true&sd=true. URL accessed on 20 August 2024.
